# Evaluation of Phytochemical, Antioxidant and Antibacterial Activity on *Astragalus Chrysostachys *Boiss. Roots

**DOI:** 10.22037/ijpr.2019.1100855

**Published:** 2019

**Authors:** Javad Ghasemian-Yadegari, Sanaz Hamedeyazdan, Hossein Nazemiyeh, Fatemeh Fathiazad

**Affiliations:** *Department of Pharmacognosy, Faculty of Pharmacy, Tabriz University of Medical Sciences, Tabriz, Iran.*

**Keywords:** Apigenin-6, 8-di-C-glucoside, Croweacin, GC/MS, DPPH scavenging, Essential oil

## Abstract

*Astragalus* is a well-known genus in Leguminosae family that represented more than 800 species growing in Iran. Nevertheless, there are a few reports on *Astragalus* plants endemic to Iran. The roots of *Astragalus* plants are rich in saponins, flavonoids and polysaccharides that possess various pharmacological activities. In the present study, chemical components, antioxidant and antibacterial activity of *Astragalus chrysostachys *Boiss*. *roots were evaluated. For determination of phytochemicals in *Astragalus chrysostachys Boiss. *roots, total hydroalcoholic extract was fractionated with ethyl acetate and n-butanol. Ethyl acetate extract as a flavonoid rich extract was analyzed using vacuum liquid chromatography and preparative TLC and consequently a major flavonoid was isolated. The structure of the obtained compound was elucidated with 1D and 2D NMR experiments. Additionally, the essential oil of the roots was analyzed by GC-MS. Antioxidant activity of all extracts was evaluated by different assays. Moreover, antibacterial activities of the extracts were also investigated against 2 Gram-positive and 2 Gram-negative bacteria using Micro-dilution Broth method. Apigenin-6, 8-di-C-glucoside was detected in ethyl acetate extract for the first time in genus *Astragalus*. In addition, m-tolualdehyde, acetophenone, croweacin were found to be characteristics of the volatile oil of roots. Ethyl acetate extracts revealed notable antioxidant activity in DPPH scavenging assay with IC50 value of 14.6 µg/mL. Evaluation of antibacterial activity on the tested extracts showed mild activity against Gram-positive bacteria. Since there have been no reports on *Astragalus chrysostachys *Boiss. to date, the present data might be promising for application of this plant derivatives in phytotherapeutic practice.

## Introduction

Since ancient times, natural medicines especially herbal medicines, has been widely used to treat or alleviate various disorders. Over the last century, development in chemistry and discovery of synthetic compounds, stem from complex synthesis methods, led to discovery of more effective medicines and replacement of ancient natural treatments with new drugs and modern pharmaceuticals. 

Even though modern medicines were able to treat many incurable and fatal diseases but herbal treatments have never been ignored. Plants, as huge natural resources of phytochemicals, have been found to exert beneficial activity in variety of diseases. 

In addition, number of herbal products and their medicinal purposes has been increased nowadays. Our country, among Asian countries, has various and fascinating medicinal plants due to its climate diversity in different regions. *Astragalus L. *is a large genus among vascular plants from Legominales tribe and Fabaceae family. The plants from this genus are generally called as Milkvetch (1). It has vast distribution in the world which mostly grows in cold, dry, and semi dry areas (2). More than 2500 species of *Astragalus *have been identified throughout the world so far. In Iran numerous species of *Astragalus *genus have been recognized which is around 800 species (3). This plant usually grows in mountainous area of Iran and is thorny. Due to its strong roots it also plays a significant role in prevention of soil erosion. Many people in Iran are familiar with *Astragalus *because of the reputation of *Persian tragacant*; a valuable polysaccharide in cosmetic industry obtained from *A. gummifera *(4).

Since ancient time, members of *Astragalus* genus, especially *A. membranaceus, A. mongholicus, A. trigonius, *and *A. gummifera* have been used as an important drug in traditional medicine in China, Russia, and Bulgaria to improve immunity system (5,6). Antibacterial and antifungal effects of *Astragalus *plants have been proved in many researches (7-10). These studies revealed the contribution of saponins in these effects. Some species of this genus showed anti-protozoal effect (11). The protective effect of *Astragalus* L. in cardiovascular system (12-14), liver (15), diabetes (16), and neurodegenerative diseases (17) suggest the presence of antioxidant components in their tissues (18). 

Down the ages, development of drug resistance pathogens has evoked interests to screen natural resources in order to introduce novel antimicrobial substances. Moreover, the adverse effect of oxidative stress, due to an excessive formation of free radicals, is another serious issue which causes important destructive effects on human health. Nowadays, medicinal plants as a natural source of antimicrobial and antioxidant constituents with limited side effects are attained special interest.

Hence (19,20), the present research was designed to analyze the phytochemical compositions of *Astragalus chrysostachys *Boiss*. *roots and also to evaluate the antioxidant and antibacterial properties of total hydroalcoholic (TE), ethyl acetate (EE) and n-butane (BE) extracts of *Astragalus chrysostachys *Boiss*. *roots*.*

## Experimental

*Chemicals*

1D and 2D NMR spectra were acquired on a Bruker Avance spectrometer (1H, 400 MHz and 13C, 100 MHz) at ambient temperature using a Topspin software pakage. CD_3_OD, 2,2-diphenyl-1-picrylhydrazyl (DPPH), quercetin, rutin, ethylenediaminetetraacetic acid (EDTA), gallic acid, Folin-Ciocalteu reagent, sodium nitroprusside, potassium hexacyanoferrate, Griess regent, and aluminum chloride all from Sigma Aldrich chemical company (USA) were used. Moler Hinton broth and Kieselgel 60 GF_254 _were from (Merck, Germany). All other chemicals also were of analytical grade.

*Micro-organisms*

Five microorganisms were used in this study, including four bacteria and one fungi. The bacteria that were used in this study consisted of two gram-positive, *Staphylococcus aureus* (ATCC 25923) and *Listeria monocytogenes* (ATCC 1163) and two gram-negative, *Escherichia coli* (ATCC 25922), and *Pseudomonas aeruginosa* (ATCC 27853). All bacteria have been obtained from Posture Institute of Iran and were MRSA clinical isolated.

*Plant material*

*Astragalus chrysostachys *Boiss*.* was collected in July 2012 from Arasbaran area in East Azerbaijan and authenticated at the herbarium of Agriculture and Natural Resources Research and Education Center, Tabriz, Iran, where a voucher specimen was deposited in the herbarium (Register No: 8303).

*Preparation of extracts and phytochemical analysis*

To prepare various extracts, 500 g air-dried roots powder were defatted with petroleum benzene and exhaustively extracted by maceration with 70% methanol (1L×4) at room temperature. Part of the total hydromethanolic (TE) extract was subjected to dryness *in vacuo* at 50 °C to give 4g dry TE and kept in refrigerator for further study. Afterwards, the methanol of the remaining TE from previous step was removed by rotary evaporator under vacuum at 50 °C. The aqueous residue was successively fractionated with ethyl acetate and finally n-butanol. The resultant extracts were dried under reduced pressure in evaporator which yielded 11.5g ethyl acetate extract (EE) and 14.3g n-butanol extract (BE). The EE was positive to Mg/HCl reagent (deep purple color was appeared), suggesting the presence of flavonoids. To detect the flavonoids, EE was subjected to vacuum liquid chromatography (VLC) on silica gel using step-wise gradient of CHCl_3_- EtOAc (10:90 to 0:100) followed by EtOAc-MeOH (95:5 to 0:100, 200 mL for each step) to obtain 22 fractions. Among those the fraction 14 eluted with EtOAc-MeOH (40:60) was further subjected to preparative TLC on silica gel glass plates (Kieselgel 60 GF_254_, 0.9mm thickness) using EtOAc:CH_3_COOH:HCOOH:H_2_O (100:11:11:26) as mobile phase to give one flavonoid (Rf=0.70, 8 mg). Detection was performed under UV-Visible light. 

Meanwhile, the essential oil of the fresh roots was prepared by hydrodistillation using Clevenger-type apparatus. 

*GC-MS analysis*

The essential oil was obtained by hydrodistillation. After 1 hr of distillation, the distillate was extracted with n-hexane and analyzed by GC-MS using a Shimadzu GC-MS-QP 5050A gas chromatograph equipped with a DB1 (methyl phenyl sylonane, 60 m x 0.25 mm i.d., 0.25 μm film thickness) capillary column. Helium was used as the carrier gas. The GC analysis was performed at the flow rate of 1.3 mL/min; linear velocity: 29.6 cm/s; Split ratio, 1:29; column temperature 2 min in 60 °C, 50-270 °C at 3 °C/min; injector temperature 250 °C, and 1 µL of volume injection of the essential oil. The MS detector parameters were set as follows: ionization potential, 70 eV; ion source temperature; 270 °C; quadrupole 100 °C, Solvent delay time 2 min, scan speed 2000 amu/s, scan range 30-600 amu, and EV voltage 3000 volts. 

The identification of compounds was performed on the basis of direct comparison of the retention indices and fragmentation patterns of the mass spectra with those reported in the literature as well as by computer matching with the Wiley 229, Nist 107, Nist 21 Library. The relative area percentages were obtained by FID without the use of correction factors, where the FID detector condition was set on a duplicate of the same column applying the same operational conditions. 

*Evaluation of antibacterial activity*

Quantitative evaluation of antimicrobial activity of EE, BE, and TE was performed according to micro dilution broth method and recommendation of CLSI (Clinical and Laboratory Standard Institute) in which MIC and MBC were determined. 

A two-fold dilution series of extracts from 128 to 1 mg/mL were prepared in DMSO and 100 µL of each concentration dispensed in 96 well microtitration plate. Then, each well was inoculated with 100 µL of suspension of bacterial inoculum in Moler Hinton broth, which was adjusted to 0.5 McFarland scale and incubated for 24 h at 35 °C. After incubation time, the lowest concentration of the extracts that inhibited growth of microorganism in wells was considered as MIC. 

To determine MBCs, the wells that showed no visible microbial growth were separately streaked on agar plates. Afterwards, the plates were incubated for 24h at 35 °C. The concentration which showed 99.9 percent with no formation of solid colony wasconsidered as MBC. Positive and negative controls were used. All the experiments were repeated 3 times.

*Assay for antioxidant activity*

The free radical scavenging capacity of the TE, EE, and BE extracts of *A. chrysostachys* roots were evaluated from the bleaching of the purple-colored methanolic solution of DPPH. Concisely, DPPH solution with concentration of 0.08 mg/100mL in methanol was prepared. The stock concentration of 1mg/mL of each extract was prepared in methanol followed by two-fold dilution series (i.e. 5×10^-1^, 2.5×10^-1^, 1.25×10^-1^, 6.25×10^-2^, 3.13×10^-2^ and 1.56×10^-2^ mg/mL).

Subsequently, 2 mL of DPPH solution was added to 2 mL of each sample and after a incubation time of 30 min at room temperature, absorbance of the mixtures were read at 517 nm (21). The mixture of equal volume of methanol and DPPH solution was served as control group. Furthermore, the quercetin was used as positive control. The experiments were performed in duplicate and the average absorption was noted for each concentration.

The free radical scavenging percentages (I%) were calculated as per the following formula:

I (%) = [(A_Control_- A_Sample_)/A_Control_] × 100

Where A_Control_ is the absorbance value of the control and A_Sample_ is the absorbance value of the sample. The curves of I% against each extract concentration were plotted and IC_50 _values were calculated. IC_50 _is the concentration of each extract inhibiting 50% of free DPPH radicals. 

*Nitric oxide radical inhibition assay*

Nitric oxide generated from aqueous sodium nitroprusside solution at physiological pH interacts with oxygen to produce nitrite ions, which could be assigned by the Griess reagent (22). Generally, scavengers of nitric oxide compete with oxygen resulting in reduced amounts of nitrite ions and extracts containing antioxidant ingredients inhibit nitrite production via entrapment of nitrite ions. For the experiment, 2.5 mL of sodium nitroprusside (10 mM) in phosphate buffered saline (pH 7.4) was mixed with 0.5 mL of different concentrations of TE, EE, and BE extracts. After incubation for 150 min at room temperature, 0.5 mL of the solutions was mixed with 1mL sulfanilic acid (0.33% in 20% glacial acetic acid). Later than 5 min, 1 mL of 0.1% naphthyl ethylene diaminedihydrochloric acid was added and allowed to stand for 30 minutes at room temperature to form chromophoricdiazo dye. The changes in color of the solutions from colorless to pink and up to deep purple were measured spectrophotometrically at 548 nm against a blank sample. Rutin was used as a positive control for comparing inhibition rate of nitric oxide radicals. Eventually, the percentages of inhibition were calculated and the results were reported as IC_50_ values, the concentration of the extract required to inhibit 50% of nitric oxide radicals.

The % inhibition was calculated as follow:

% Inhibition of nitric oxide radical = [A_0_-As]/A_0_×100

Where A_0_ is the absorbance before reaction with Griess reagent and As is the absorbance after reaction with Griess reagent. 

*Reducing power assay*

The Fe^3+ ^reducing power of TE, EE, and BE were assessed according to the method of Yen and Chen (23). Different concentrations of the extracts and rutinas the standard were mixed with 2.5 mL of phosphate buffer (pH 6.6) and 2.5 mL of 1% potassium ferricyanide [K_3_Fe (CN)_ 6_] solution. The mixture was incubated at 50 °C for 20 min and then 2.5 mL of trichloroacetic acid (10%, W/V) was added to stop reaction. The upper layer of the solution (2.5 mL) after centrifugation was mixed with 2.5 mL distilled water and 0.5 mL FeCl_3_ 0.1% w/v. Finally, the absorbance of the reaction mixture was measured at 700 nm spectrophotometrically.

*Metal chelating activity assay*

Chelating reaction of Ferrozine with Fe^2+^ results in formation of a red color complex. Presence of other chelating substances decreases the production of red color Ferrozine-Fe^2+^

chelate and subsequently decreases in intensity of red color. Hence, measurement of the color indicates the chelating activity of a sample to compete with ferrozine for Fe^2+^. Herein, the ability of tested extracts (TE, EE and BE) to chelate ferrous iron, were measured by little modification to Dinis method (24). Briefly, 5mL of each extract in different concentrations were mixed with 0.1 mL of 2mM of FeCl_2_ (2 mM) and 1.6 mL of deionized water was added. Later, 1mL of ferrozine (5 mM) was added and incubated for 10 min at room temperature reaching the equilibrium. After all, absorbances of the mixtures were measured at 562 nm. The percentage of inhibition of ferrozine-Fe^2+^ complex was calculated from [(A_Control_/A_Sample_)/A_Control_] × 100, where A _Control_ was the absorbance of the control (blank, without extract) and A _Sample_ was the absorbance in the presence of the extract. EDTA was used as a positive control which chelates to Fe^2+^ in a fairly large amount. Besides, the IC_50_ value, the concentration of each extract required to inhibit the formation of Fe^2+^–Ferrozine complex in 50% was calculated.

## Results

*Phytochemical analysis of EE*

**Figure 1:**
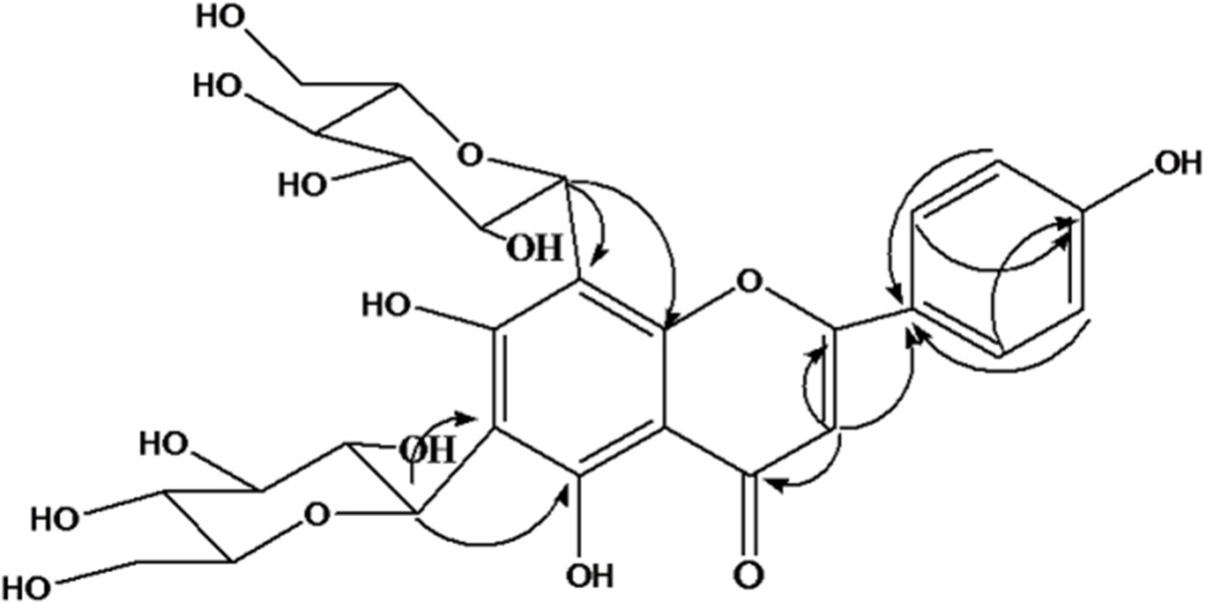
key HMBC correlation of Apigenin-6,8-di-C-glucoside

Phytochemical analysis of EE led to isolation of the predominant component of this extract. The structure elucidation was conducted with the UV, ^13^C NMR, ^1^H NMR, HMBC, and DEPT experiments. The isolated compound, yellow amorphous powder, and UV spectrum (MeOH) showed absorption maxima at 272nm and 334nm suggestingthe compound to be a flavonoid which was further confirmed with NMR spectra. In H NMR (CD_3_OD, 400MHz) spectrum two ortho-coupled doublets with integration of two protons at δ_H_6.94 (J=7.2 Hz, H-3´, 5´) and δ_H_8.02 (J=7.2Hz, H=2´,6´) indicatingthe 4´substituted B ring. The lack of signals corresponding to A ring revealed the presence of tetrasubstituted A ring moiety (Table 1). Additionally, a singlet 1H at δ_H_6.77 attributed to H-3 C ring. ^13^C NMR (CD_3_OD, 100MHz) revealed 27 carbons from which 15 carbons were assigned for a flavonoid aglycone and 12 carbons were assigned for two hexose sugar moieties. The DEPT spectrum exhibited nine quaternary carbons, five methine carbons, and one carbonyl carbon. The 1H and 13C NMR values for all the carbons were further assigned on the basis of HMBC correlations as shown in Figure 1. Based on the spectral evidences the structure of this compound was identified as Apigenin-6,8-di-C-glucoside and confirmed the comparison with literature data.

*GC-MS analysis*

The GC–MS analysis of the essential oil from the roots of* A. chrysostachys* Boiss. led to the identification of 8 compounds, representing 76.1% of the essential oil. The identified compounds are given in Table 2 in the order of their elution from the DB1-MS column and the retention indices.

*Antibacterial activity A. chrysostachys Boiss*

The results of antimicrobial screening of TE, EE, and BE on gram-positive and gram-negative bacteria were shown in Table 3. All the extracts had antibacterial activity against *S. aureus* and *L. monocytogenesis* with MIC values between 2-4 mg/mL. None of the extracts showed antibacterial activity on the* E. coli*microorganism. EE and TE were also inactive towards *P. aeroginosa.* Table 3 shows inhibitory and bactericidal concentration of various extracts.

*In-vitro antioxidant activity*

**Figure 2:**
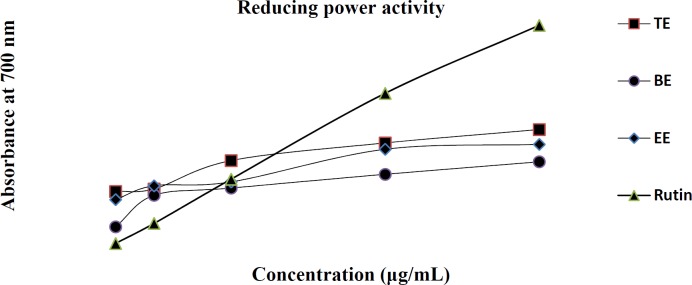
Reducing power of TE, EE and BE compared to the positive control of rutin at different concentrations

Various *in-vitro* antioxidant assays on the TE, EE, and BE were accomplished and the established data for DPPH and Nitric oxide radical scavenging activities along with metal chelating activity were shown in table 4. Additionally, data for reducing power activity had been exhibited in Figure 2.

Among the three tested extracts for *in-vitro *antioxidant activity, EE exhibited potent antioxidant activity in DPPH and moderate activity in nitric oxide radical scavenging assays with the IC_50_ values 14.6 and 730.7µg/mL, respectively (Table 4). Nonetheless, EE were inactive in metal chelating assay. TE exhibited a relatively higher IC_50_ value than for the standard of EDTA for metal chelating activity with an IC_50_ value of 268.9 µg/mL. In the case of TE, an IC_50_ of 54.1µg/mL was established for DPPH radical scavenging and 268.9µg/mL for metal chelating assay (Table 4). BE showed almost presentable antioxidant activities in DPPH and metal chelating assays with the IC_50_ values 51.7 and 213.7 µg/mL, correspondingly.

The reducing capacity of a compound might be a pointer of its potential antioxidant activity.For this purpose we investigated the Fe^3+^–Fe^2+^ transformation in the presence of different sample extracts. As shown in Figure 2, Fe^3+^ was transformed to Fe^2+^ in the presence of TE, EE, and BE and the reference compound rutin to measure the reductive capability. Although, the absorbances of the extracts and rutin were almost the same at lower concentrations (50 µg/mL), it was shown that at higher concentrations of 600 µg/mL, TE, EE, and BE the absorbances were around 0.5 and 0.7, compared to the absorbance of 1.3 for control of rutin, exhibiting lower reducing activity than the control rutin. The results indicated that maximum activity was shown at higher doses by rutin.

**Table 1:** 1H and 13C NMR data of aglyconeofApigenin-6,8-di-C- glucoside in CD3OD; (δ in ppm, *J *in Hz).

4			
**Position**	**δH**	**δC**	**DEPT**
**1**	-	-	-
**2**	-	164.35	*Q
**3**	6.77s	102.89	CH
**4**	-	182.5	-C=O
**5**	-	155.6	*Q
**6**	-	108	*Q
**7**	-	161.78	*Q
**8**	-	104.45	*Q
**9**	-	159.21	*Q
**10**	-	105.7	*Q
**1'**	-	121.91	*Q
**2'**	8.02(d, *J*=7.2)	129.4	CH
**3'**	6.95(d, *J*=7.2)	116.38	CH
**4'**	-	161.23	*Q
**5'**	6.95(d, *J*=7.2)	116.38	CH
**6'**	8.02(d, *J*=7.2)	129.4	CH
**1''**	4.78	75.24	CH
**2''**	3-4**	71.54	CH
**3''**	3-4**	79.25	CH
**4''**	3-4**	70.95	CH
**5''**	3-4**	81.25	CH
**6''**	3-4**	61.58	CH2
**1'''**	4.95	77.15	CH
**2'''**	3-4**	71.69	CH
**3'''**	3-4**	78.32	CH
**4'''**	3-4**	70.67	CH
**5'''**	3-4**	82.28	CH
**6'''**	3-4**	61.73	CH2

**Table 2:** Chemical composition of the essential oil from roots of *A. chrysostachys *Boiss

**No.**	**Compounds**	**Percentage**	**Formula**	**Molecular weight**	**RI** *	**Identification Method**
**1**	Acetophenone	16.2	C8 H8 O	120	-	GC/MS
**2**	m-Tolualdehyde	29.7	C8 H8 O	120	-	GC/MS
**3**	Linalool	2.4	C10 H18 O	154	-	GC/MS
**4**	Undecane	0.4	C11 H24	156	1164	GC/MS, RI
**5**	Dodecene	9.9	C12 H24	168	1255	GC/MS, RI
**6**	Croweacin	12.3	C11 H12 O3	192	1332	GC/MS, RI
**7**	Carotol	1.5	C15 H26 O	222	1497	GC/MS, RI
**8**	Hexahydrofarnesyl acetone	3.7	C18 H36 O	268	1768	GC/MS, RI
**Total identified**		76.1(%)				

* RI is the Retention Index relative to C10–C20 n-alkanes on the DB-1 column.

**Table 3:** Minimum inhibitory and bacteriostatic concentrations of different extracts of *AstragaluschrysostachysBoiss.*roots against some bacterial strains

	***S. aureus***		***L. monocytogenesis***		***P. aeroginosa***		***E. coli***	
	**MIC**	**MBC**	**MIC**	**MBC**	**MIC**	**MBC**	**MIC**	**MBC**
BE (mg/mL)	4	8	4	-	4	-	-	-
EE(mg/mL)	4	-	2	-	-	-	-	-
TE (mg/mL)	4	-	2	-	-	-	-	-
Penicillin (µg/mL)	2.5	2.5	-	-	-	-	-	-
Ciprofloxacin (µg/mL)	0.25	2	0.12	0.12	0.1	0.3	0.47	0.94
Gentamicin (µg/mL)	2	-	-	-	0.31	0.31	1	2.5
								

TE: Total hydroalcoholic extract, EE: Ethyl acetate extract, BE: n-Butanol extract, MIC: Minimum inhibitory concentrations, MBC: Minimum bacteriostatic concentrations

-: No antibacterial activity

**Table 4:** Effect of TE, EE and BE on DPPH and nitric oxide radical scavenging along with metal chelating activity (IC50 values) with their standards

**Activity**	**Extract / Standard**	**IC** **50**
DPPH radical scavenging	TE	54.1 µg/mL
	EE	14.6 µg/mL
	BE	51.7 µg/mL
	Quercetin	3.0 μg/mL
Nitric oxide radical scavenging	TE	-
	EE	730.7 µg/mL
	BE	-
	Rutin	140.1 µg/mL
Metal chelating	TE	268.9 µg/mL
	EE	-
	BE	213.7 µg/mL
	EDTA	1.2 µg/mL

## Discussion

In recent decades, phytochemical investigation on *Astragalus* genus has been extensively conducted in depth. To the best of our knowledge, substantial evidences indicated triterpenesaponins, flavonoids and polysaccharides are the most important metabolites in this genus. Moreover, numerous studies on pharmacological activity on these plants uncovered the exclusive capacity of *Astragalus* plants in protection human health against fatal diseases, like; immunodeficiency, cardiovascular disorders, hepatic injury, cancer, diabetes and neurodegenerative, and inflammatory diseases (25-27). In this regard, there is ample evidence indicating that the mentioned metabolites are the main contributors in pharmacological effects of *Astragalus* plants (27-31). Therefore, it is of great importance to investigate on various *Astragalus *species either from phytochemical or bioactivity point of view. In the present study, phytochemical analysis of *A. chrysostachys *roots led to isolation of Apigenin-di-*C*-glucoside as a major flavonoid in ethyl acetate extract. In previous studies, Apigenin and Apigenin-*O*- glycosides were reported in *A.propinquum*, *A. bombycinus*, and *A. verrucosus*. Apigenin-8-*C*-glucoside (Vitexin) was also found in *A. corniculatus *(1). It is worth mentioning that this is the first report on isolation of Apigenin-di-*C*-glucoside from genus *Astragalus.*

Results from analysis of GC-MS revealed that the *A. chrysostachys* essential oil was characterized mainly by the presence of m-tolualdehyde (29.7%), acetophenone (16.2%), croweacin (12.3%) and dodecene (9.9%). While the chemistry of *Astragalus* species have been studied extensively, studies on the volatile components of *Astragalus* plants have not been numerous. Platikanov *et al*. investigated on volatile components of the aerial parts of four *Astragalus *species. The authors reported complex composition of volatile compounds including alcohols, aldehydes, ketones, acids, esters, ethers, hydrocarbons, terpenes, and chlorinated compounds (32). It was also reported by Movafeghi *et al*. that the essential oil of the leaves and roots of *A. microcephalus* and *A. compactus* contained pentanal 3-methyl, octadecane, caryophyllene, and toxic chlorinated compounds (33,34). Our results were generally similar, except the toxic chlorinated compound which was absent among volatile constituents of *A. chrysostachys* roots.

The antibacterial assay for TE, EE, and BE exhibited that *S. aureus* and *L. monocytogenesis* were susceptible to the extracts to some extent; however the antibacterial potency were negligible in comparison to the standards of penicillin and ciprofloxacin. 

Oxidation process is an essential and vital metabolic reaction in the cells and body. The production of free radicals through these reactions plays a major role in various normal regulatory systems in the body. Despite the dependence of the body to oxidative metabolism, an excess formation of free radicals cause oxidative stress leading to destructive effects on cells and multiple degrees of the cell damage. There is ample evidence indicatingthat free radicals are involved in the large number of diseases including cancer, alzheimer, cardiovascular problems, diabetes, atherosclerosis, aging process, rheumatoid arthritis and Parkinson’s disease. Therefore, naturally occurring antioxidants has drawn the great attention of researchers for their potential in prevention of such diseases.

According to the results of antioxidant activity for *A. chrysostachys, *it was observed that all the tested extracts revealed admissible DPPH scavenging activity with increasing extract concentrations. Nonetheless, it was perceptible that TE, EE and BE did not show convenient reductive potential in reducing power assay, hence, it could not serve as electron donor. In the case of nitric oxide scavenging only EE showed slight activity where percentage of inhibition increased via increasing EE concentration. Into the bargain, plant material counteracting the effect of nitric oxide formation in human body might also be beneficial in prohibition of the excessive generation of nitric oxide implicated in inflammatory conditions, cancer, etc (35).

Likewise, metal chelating activity is of use in reducing oxyradicals and lipid peroxidation bringing about diminished oxidative damages like cardiovascular diseases (36). In the presence of other chelating agents TE and BE fairly disrupted ferrozine complex formation, suggesting their moderate antioxidant activity in relation to the iron binding capacity. 

Taken as a whole, TE, EE and BE exhibited different degrees of antioxidant activity in the entire studied models. Further evaluation of particular compounds both *in-vitro* and *in-vivo* antioxidant assays is essential to determine their mechanism of action.

Even though more detailed phytochemical examination is mandatory, our findings as a first report dealing with phytochemical analysis of *A. chrysostachys*, confirmed that flavonoids were the prevailing group of compounds in EE. Justifying the striking antioxidant activity of this plant, we might suggest *A. chrysostachys* as a natural resource for phytotherapeutic purposes
